# Interaction of micron and nano-sized particles with cells of the dura mater

**DOI:** 10.1002/jbm.b.33129

**Published:** 2014-03-06

**Authors:** Iraklis Papageorgiou, Rainy Marsh, Joanne L Tipper, Richard M Hall, John Fisher, Eileen Ingham

**Affiliations:** 1 Institute of Medical & Biological Engineering (IMBE), Faculty of Biological Sciences, University of LeedsLeeds, LS2 9JT, UK; 2 Institute of Medical & Biological Engineering (IMBE), School of Mechanical Engineering University of LeedsLeeds, LS2 9JT, UK

**Keywords:** total disc replacement, meninges, dura mater, phagocytosis

## Abstract

Intervertebral total disc replacements (TDR) are used in the treatment of degenerative spinal disc disease. There are, however, concerns that they may be subject to long-term failure due to wear. The adverse effects of TDR wear have the potential to manifest in the dura mater and surrounding tissues. The aim of this study was to investigate the physiological structure of the dura mater, isolate the resident dural epithelial and stromal cells and analyse the capacity of these cells to internalise model polymer particles. The porcine dura mater was a collagen-rich structure encompassing regularly arranged fibroblastic cells within an outermost epithelial cell layer. The isolated dural epithelial cells had endothelial cell characteristics (positive for von Willebrand factor, CD31, E-cadherin and desmoplakin) and barrier functionality whereas the fibroblastic cells were positive for collagen I and III, tenascin and actin. The capacity of the dural cells to take up model particles was dependent on particle size. Nanometer sized particles readily penetrated both types of cells. However, dural fibroblasts engulfed micron-sized particles at a much higher rate than dural epithelial cells. The study suggested that dural epithelial cells may offer some barrier to the penetration of micron-sized particles but not nanometer sized particles. © 2014 The Authors. Journal of Biomedical Materials Research Part B: Applied Biomaterials Published by Wiley Periodicals, Inc. J Biomed Mater Res Part B: Appl Biomater, 102B: 1496–1505, 2014.

## INTRODUCTION

Total intervertebral disc replacement (TDR) is used for the treatment of degenerative disc.^1^–^5^ The implant materials are the same as those used in the hip and knee prostheses^2^ either metal-on-polyethylene (Charité, De Puy-J&J; Prodisc L, Spine Solutions) or metal-on-metal (Maverick disc, Medtronic Sofamor Danek).^5^ Between 1984 and 2004 around 9000 patients received Charité implants, mostly in Europe. The success of the Charité TDR remains controversial due to a broad range of outcomes.^6^ Lemaire et al.^7^ reported excellent or good clinical outcomes in 90% of cases at 10-years, however, Pultzier et al.^8^ reported 60% ankylosis at 17 years. A later study (10 years follow-up) reported good clinical outcome in 82% of 106 patients.^9^ The Maverick disc has been used clinically since 2002^10^ and at short term follow up (2 years) 64 patients had a degree of mobility close to normal and low postoperative complications.^11^ However, one case report indicated extensive metallosis and osteonecrosis at one year in a well-positioned device with no visible loosening on radiographic analysis.^12^

The issue of wear particle generation over time in TDR requires attention. Polyethylene wear debris is known to cause osteolysis and aseptic loosening in total hip and knee replacements. There are concerns that wear particles will also cause problems in the spine. Polyethylene wear particles of 0.2 to 10 microns in size are potent stimulators of mononuclear phagocytes and induce the release of proinflammatory cytokines that activate osteoclasts leading to bone resorption.^13^ Wear particles generated by metal-on-metal articulations are in the nanometer size range^14^ and there are concerns relating to their toxicity, potential to cause local tissue necrosis and immunological reactions.^15^ In hip and knee implants the wear debris has the ability to disseminate systemically beyond the periarticular tissue.^16^

There are increasing reports of dissemination of wear debris and biological reactions associated with TDRs. Polyethylene and metal particles have been detected within the surrounding tissues 12 weeks following implantation of a cervical TDR (Prodisc C), with no associated inflammatory reaction.^17^ However, at longer implantation times (∼8 years), polyethylene particles have been associated with chronic inflammatory reactions. A positive correlation between the number of polyethylene particles and the number of macrophages and giant cells detected in the periprosthetic tissue has been reported at revision surgery.^18^ The concentration of cobalt and chromium ions, in the blood of the patients with metal-on-metal TDRs has been reported to be significantly higher than controls and reached levels observed in total hip implants.^19^ In one case report, metal wear debris caused the development of a soft tissue mass posterior to the implanted disc that encroached the spinal cord.^20^ Similarly, Berry et al.^21^ reported, in a case study that the presence of metal particles caused the development of a granulomatous mass at the level of the Maverick disc prosthesis 3 years following implantation, with clinical implications such as spinal stenosis and iliac vein thrombosis.

It is likely that the wear particles that are produced in TDR interact with the dura mater (meninges). Therefore there is a need to understand how particles interact with the cells of the dura mater and the effect of particle size on these interactions. Here, we utilized a porcine model system. Dura mater was acquired through aseptic dissection of pigs and analyzed by histology and immunohistochemistry. The resident epithelial and stromal fibroblast cells, were purified, expanded in culture and characterized by immunofluorescence. The cell lines were then exposed to polystyrene micron- and nano-meter sized particles and their phagocytic ability^22^ was determined using confocal and deconvolution microscopy.

## MATERIALS AND METHODS

### Isolation of the dura mater from pigs

Pigs from the University of Leeds farm (Large White females, 65 kg) were humanely killed using a UK Home Office procedure. Part of the vertebral column (thoracic region, T2-T10) was aseptically dissected and transferred to the laboratory. The spinal cord with the meninges attached was dissected and incubated in a solution of Gentamicin (Sigma, UK) 50 ng mL^−1^ and Nystatin (Sigma, UK) 100 U mL^−1^ in phosphate buffered saline (PBS; Sigma, UK) for an hour at room temperature.

### Tissue processing

Tissue samples [1 cm^2^] were fixed in 10% (v/v) neutral buffered formalin for 48 h, processed and embedded in paraffin wax and sectioned at 4 to 10 μm (RM2125RTR, Leica Microsystems). Sections were either stained using standard haematoxylin and eosin or used for immunohistochemical staining. Tissue samples were also snap-frozen in liquid nitrogen, embedded in optimal cutting temperature (OCT) medium (Fisher) and sectioned (6 µm) using a cryostat (CM1850, Leica Microsystems). Sections were air dried for 30 min and stored at −80 C.

### Immunohistochemical analysis of the dura mater

Selected extracellular matrix proteins and cell markers were identified using the following antibodies at the dilutions given: collagen I (D58-G9, IgG 1k, 1:100, Milipore), collagen II (COL-II, IgG 1k, 1:1000, Milipore,), collagen III (IE7-D7, IgG1, 1:50, Milipore,), fibronectin (568, IgG1, 1:2000, Vector), laminin (LAM-89, IgG1, 1:100, Sigma), vimentin (VIM3B4, IgG2a, 1:2000, Novocastra), von Willebrand factor (VWF; rabbit immunoglobulin fraction, 1:1000, Dako), integrin 1β (JB1B, IgG2a, 1:100, AbD Serotec), desmoplakin (DP2.15, IgG1, 1:200, AbD Serotec), and E-cadherin (36B5, IgG1, 1:100, Vector). Antibody to collagen III and E-cadherin were used with cryosections and all of the remaining antibodies were used with paraffin wax-embeded sections. For laminin, fibronectin, vimentin and collagen II, antigens were retrieved using proteinase K digestion. For collagen I, VWF, integrin 1β antigens were retrieved by trypsin digestion using standard protocols. Sections were rehydrated and blocked in 0.6% (v/v) H_2_O_2_ (Sigma) for 10 min and washed three times with Tris-buffered saline [TBS 50 m*M* Tris (Sigma), adjusted to pH 7.6 using HCl (VWR), 150 m*M* NaCl (VWR) in distilled water]. Bound antibodies were detected using the EnVision+ Dual Link System-HRP (DAB+) kit. Sections were counterstained with haematoxylin (Bios Europe Ltd). Histology and immunohistochemical images were captured using an upright microscope (Olympus BX51) fitted with a digital camera (Olympus XC50) and processed using AnalySIS Image Processing software.

### Isolation of cells from the dural membrane (dura mater)

Samples (∼1 cm^2^), of dural membrane were dissected aseptically away from the arachnoid and the pia mater (avoid vascular endothelial contamination) and placed in a six-well plate (Thermo Fisher Scientific Ltd), and cultured in medium m199 (Sigma) supplemented with foetal bovine serum (20% v/v, Lonza), l-glutamine (2 m*M*, Lonza), sodium pyruvate (1.1 mg mL^−1^, Sigma), heparin (10 U mL^−1^), penicillin/streptomycin (50 U mL^−1^, Lonza) and endothelial growth factor (15 µg mL^−1^, Sigma) at 37°C in 5% (v/v) CO_2_ in air. After 7 days of outgrowth, the cells were harvested and transferred to 75 cm^2^ flasks (Fisher) and expanded in supplemented m199 medium.

### Separation of dural fibroblasts and epithelial cells

Cells were suspended in PBS with 0.1% w/v bovine serum albumin (Sigma) and separated using anti-CD-31 labeled magnetic Dynabeads® (Endothelial cell-specific antibody, Invitrogen) according to the manufacturer's instructions. This was carried out twice. Both dural epithelial and fibroblast cells were expanded and a bank of cells created.

### Cell phenotyping

In addition to those listed above the primary antibodies used were: anti-fibronectin (rabbit immunoglobulin fraction, 1:100, Dako), anti-tenascin (TN2, IgG1k, 1:200, Novocastra), anti-collagen III (IgG1, 1:25, Chemicon), anti-actin α-smooth muscle (1A4, IgG2a, 1:200, Sigma), anti-E-cadherin (36B5, IgG1, 1:25, Vector), anti-human CD-31 (9G11, IgG1, 1:20, R&D systems), anti-desmoplakin I + II (2Q400, IgG1, 1:50, Abcam), anti-glucose transporter 1 (IgG, 1:50, Abcam), anti-porcine endothelial cells (MIL11, IgE, 1:100, AbD Serotec), anti-human fibroblast/epithelial cells (D7-FIB, IgG2a, 1:100, AbD Serotec), anti-smooth muscle myosin heavy chain (N1/5, IgG1, 1:100, Chemicon), anti-smoothelin (IgG1, 1:100, Millipore), anti-desmin (DE-R-11, IgG1, 1:200, Vector).

Cells were cultured on multitest slides (eight-well, MP Biomedics) for 24 h, fixed in ice-cold methanol:acetone (1:1), air-dried and soaked sequentially in dH_2_O and 0.05% (w/v) saponin (Sigma) in TBS. Primary antibody or isotype control (50 µL) was added and the cells were incubated for 1 h at room temperature, washed three times with TBS and incubated with fluorescein labeled secondary antibody [anti-mouse (goat, F(ab)_2_ fragment, Invitrogen) or anti-rabbit (goat, F(ab)_2_ fragment, Invitrogen)] for 30 min in the dark. Cells were washed with TBS and counterstained with Hoechst solution (1 µg mL^−1^; Sigma) for 10 min. The slides were then examined by fluorescence microscopy (Olympus, BX51) and images were captured as above.

### Particle characterization

Polystyrene FluoSpheres of a nominal 1 µm and 40 nm size were purchased from Invitrogen, UK. These were ultraclean polysterene microspheres FITC-labeled and their sizes were determined by field emission gun scanning electron microscopy (FEG-SEM) to be 1.07 ± 0.012 µm and 54 ± 13 nm, respectively.

### Cellular uptake of the particles

Porcine dural epithelial and fibroblast cells were seeded onto multitest microscope slides (8-well, MP Biomedics) at 5 × 10^3^ cells per spot and incubated at 37°C in 5% (v/v) CO_2_ in air overnight. The nanometer and micrometer-size particles were sonicated in an ultra-sonication water bath (Grant Instruments Ltd) for 15min. A 1% (w/v) suspension (50 µL) was added to the cells. The suspensions of 40nm and 1µm sized particles contained 5.68 × 10^11^ and 3.6 × 10^7^ particles mL^−1^ respectively (numbers as specified by company, Molecular Probes/Invitrogen,UK). The particle exposed cells were incubated for 1, 2, and 3 days with 3 replicates per time point. At each time point the cells were washed twice with PBS to remove any medium and any unbound particles, fixed in ice-cold methanol:acetone (1:1) for 2 min, rehydrated and immersed in 0.05% (w/v) saponin in TBS. Rhodamine-phaloidin (Invitrogen) solution (0.66 µ*M*) was then added for 1h and the cells were washed three times with TBS and counterstained with Hoechst solution (1 µg mL^−1^) for 10 min or with Sytox-Green (0.5 m*M*; Invitrogen) for 30 min. Slides were washed with TBS and dH_2_O and mounted in Dabco/glycerol.

Cells were visualized as described above. Images of 100 cells per slide were processed with AnalySIS Image Processing software. This was repeated using three replicate cultures per time point. The percentage of cells internalising particles at each time point was blind-scored by manual counting from an independent observer. The cells were separated into five categories depending on the number of particles that had been internalized (0, 1 to 5, 6 to 10, 11 to 20, more than 21 particles per cell). Data were arcsin transformed for calculation of 95% confidence limits, analysis by one way ANOVA and calculation of minimum significant difference (*p* < 0.05) between means using the T-method. Data were back transformed to percentages for presentation.

Cells were also visualized by confocal microscopy (Upright Zeiss LSM 510 META Axioplan 2) and using a Delta Vision 3D Digital Deconvolution Restoration System based around an Olympus IX70 inverted microscope, with SoftWorx deconvolution software. A stack of images was acquired to generate a video of the cells and their interaction with the fluorescent particles in three dimensions.

## RESULTS

### Histology/immunohistochemical analysis of porcine meninges

**Figure 1:**
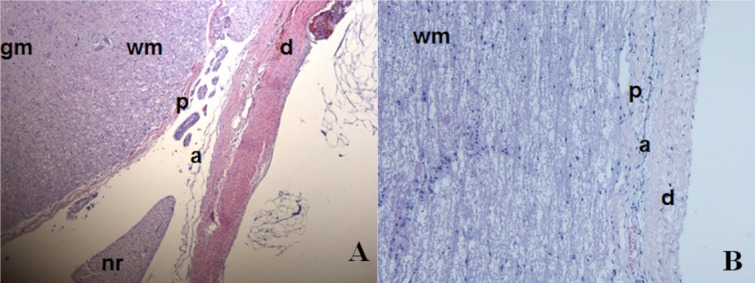
Haematoxylin and Eosin stained porcine meninges. (A) Transverse view of the 3 meninges. (B) Longitudinal view of the meninges. gm—Grey matter of spinal cord, wm—white matter of spinal cord, p—Pia mater, nr—nerve root, a—arachnoid mater, d—dura mater. [Color figure can be viewed in the online issue, which is available at wileyonlinelibrary.com.]

The structural complexity of the porcine meninges is depicted in Figure 1. The dura mater was a dense membranous structure that surrounded the more porous and highly vascularized arachnoid mater. This looser structure surrounded a thinner membrane covering the spinal cord, the pia mater [Figure 1(A)]. In the longitudinal view the layers appeared more tightly packed [Figure 1(B)].

**Figure 2:**
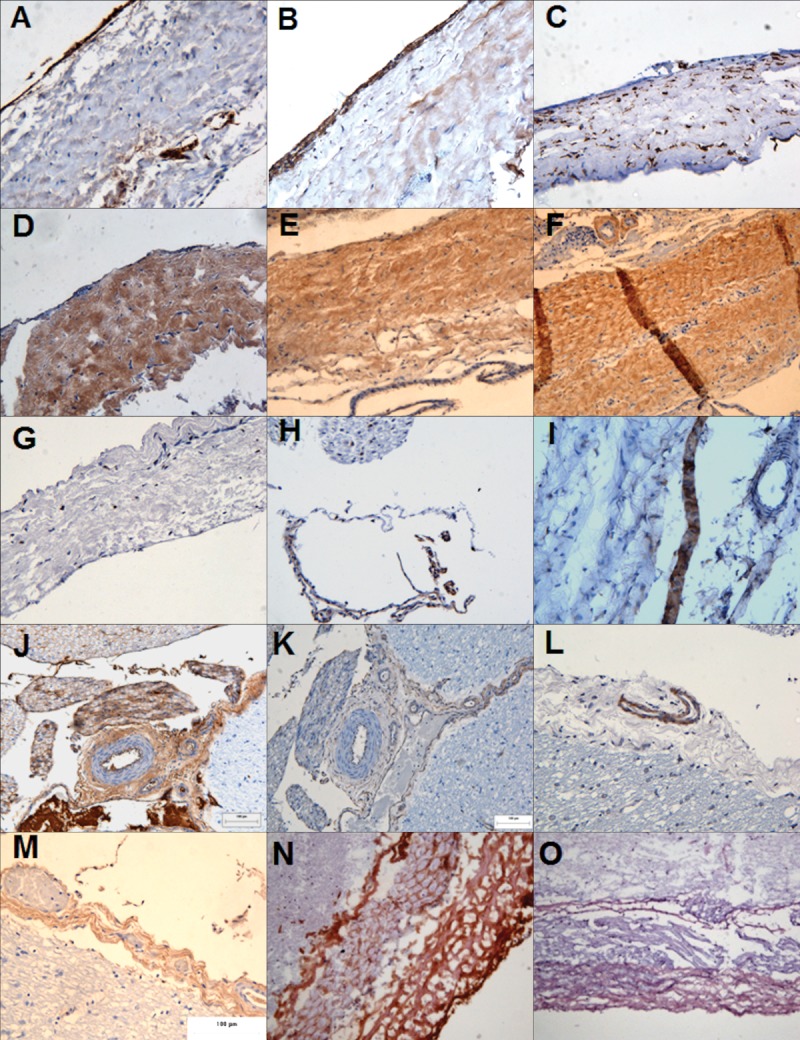
Immunohistochemical analysis of the porcine meninges. The meninges composed of the dura mater (A–G), the arachnoid mater (H–I) and the pia mater (J–M) in formalin fixed tissue. The porcine spinal cord was cryosectioned and the porcine dura mater (N–O) was stained. The tissue sections were labeled for: (A) von Willebrand factor, (B and K) fibronectin, (C and J) vimentin, (D) laminin, (E) collagen I, (F and M) collagen II, (G, H, and L) integrin 1β, (I) Desmoplakin, (N) collagen III, (O) E-cadherin. Images were captured at ×200 magnification. [Color figure can be viewed in the online issue, which is available at wileyonlinelibrary.com.]

The dura mater (Figure 2) had an outer layer of epithelial cells that stained positively for VWF and fibronectin and negative for vimentin and laminin. The inner layer contained fibroblastic cells which were vimentin, fibronectin, laminin, and integrin-1β positive and VWF negative. These fibroblast cells were scattered through a matrix of predominantly parallel collagen I and II fibers which was vascularised. In the arachnoid mater, the cells expressed integrin 1β and desmoplakin. In the pia mater, the cells strongly expressed vimentin, weakly expressed fibronectin and were negative integrin 1β. Pia mater contained a rich meshwork of predominantly collagen II fibers and was more vascularized than the dura mater with vessel walls stained positive for integrin1β, collagen I & III fibers, and VWF (images not shown).

Antibody staining of cryosections (Figure 2) supported the above findings with a few additions. The dural collagen matrix was composed not only of collagen I and II fibers but also of collagen III fibers. The epithelial layer also expressed the trans-membrane protein E-cadherin. These findings are summarized in Table1.

**Table 1:** Expression of Extracellular Matrix and Cellular Proteins in the Porcine Meninges

	Dura mater			Arachnoid mater		Pia mater		Pial vasculature
	ECM	EC	Fibs	ECM	Cells	ECM	Cells	
Laminin	+++	−	−	+	−	++	−	−
Collagen I	+++	+	−	+	−	++	+	+++
Collagen II	+++	−	−	+	−	++	−	−
Collagen III	+++	−	+/−	+	−	++	−	+++
Fibronectin	+	+++	−	+++	−	+++	−	−
Von Willebrand factor	−	+++	−	−	−	−	−	+++
Integrin 1β	−	−	+	−	+	−	−	++
Desmoplakin	−	−	−	−	+++	−	−	−
Vimentin	−	−	+++	−	++	−	++	−

The intensity of the immunohistochemical staining was categorized as follows: +: low expression, ++: moderate expression, +++: strong expression, −: no expression. ECM: extracellular matrix, EC: epithelial cells, Fibs: fibroblast cells.

### Cell morphology, separation, and phenotyping

Primary porcine dural tissue explants cells comprised a mixture of epithelial and fibroblastic cells. After magnetic bead separation, the cells were separated into fibroblastic and epithelial cells. Both cell types were positive for fibronectin, tenascin, actin, collagen I and III (Table2). The dural epithelial cells showed characteristics of typical endothelial cells since they were positive for VWF and CD31. VWF staining was localized to small intracellular granules. CD31 was localized to the cell membrane and when visualized at high magnification punctate staining was apparent. The dural epithelial cells, but not fibroblasts were positive for the transmembrane protein E-cadherin. In order to identify any potential for barrier formation, cells analyzed for desmoplakin I/II and glucose transporter (Glut1) expression. Dural epithelial cells, but not fibroblasts expressed desmoplakin I/II, especially in regions with high cellular confluency. Both cell types expressed Glut1. Neither dural epithelial nor fibroblast cells expressed smooth muscle markers (Table2) indicating a lack of smooth muscle cell contamination.

**Table 2:** Expression of Proteins by Dural Epithelial and Fibroblast Cells as Determined by Indirect Immunofluorescence

Antibody specificity	Epithelial cells	Fibroblasts cells	Primary smooth muscle cells
Fibronectin	+ve	+ve	-------
Tenascin	+ve	+ve	-------
Collagen I	+ve	+ve	--------
Collagen III	+ve	+ve	--------
Actin	+ve	+ve	+ve
Glucose transporter (Glut-1)	+ve	+ve	+ve
Von Willebrand factor	+ve	−ve	−ve
E-Cadherin	+ve	−ve	---------
CD-31	+ve	−ve	−ve
Desmoplakin	+ve	−ve	−ve
Porcine endothelial cells	−ve	−ve	−ve
Human fibroblast/epithelial cells	−ve	−ve	-------
Smooth muscle myosin, Heavy chain	−ve	−ve	+ve
Smoothelin	−ve	−ve	+ve
Desmin	−ve	−ve	+ve

Primary smooth muscle cells were used as a positive control for smooth muscle cell contamination (+ve: positive staining, −ve: negative/no staining, ------: not tested).

### Cellular uptake of the particles

**Figure 3:**
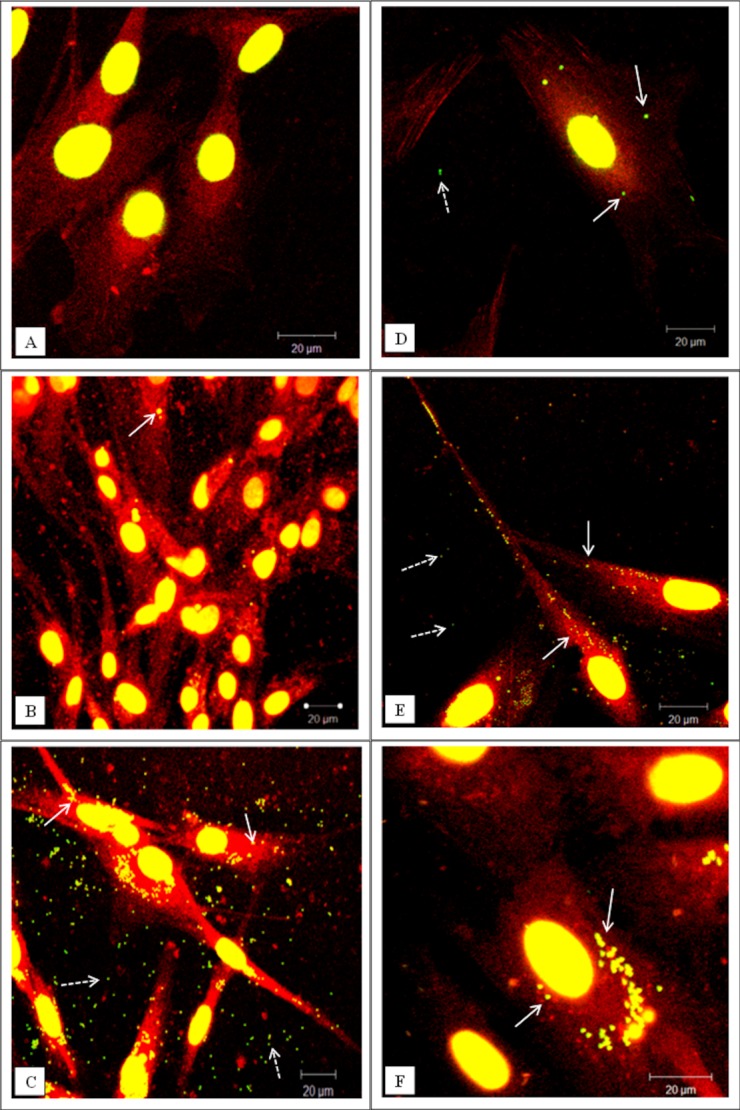
Representative confocal microscopy images of cells exposed to green fluorescent microspheres (1 µm in diameter). (A–C) Dural epithelial cells. (D–F) Dural fibroblasts. (A, D) Cells exposed for 1 day. (B, E) Cells exposed for 2 days. (C, F) Cells exposed for 3 days. White arrows indicate particles internalized whereas white dashed arrows indicate particles outside the cells. The actin filaments were stained with rhodamine phalloidin and the nucleus with sytox green. All the images were taken at 630× magnification. In total 300 cells were imaged (three replicates × 100) at each time point. [Color figure can be viewed in the online issue, which is available at wileyonlinelibrary.com.]

The capacity of the dural cells to internalize micron (1 µm) and nano-sized (40 nm) particles was assessed over a period of 1 and 3 days. Representative examples of particle uptake are shown in Figures 3 and Figure 5 for the micron and nano-sized particles respectively.

In the case of fluorescent micron-particles, the dural epithelial cells did not readily internalise significant numbers of particles over a period of 24 h [Figure 3(A)]. However, for the same exposure period the fibroblasts internalized substantial numbers of particles [Figure 3(D)]. The inability of the micron-particles to enter the epithelial cells was a temporal phenomenon and after 2 days some particles were internalized [Figure 3(B)]. At the same exposure time, the fibroblasts had internalized large numbers of particles [Figure 3(E)]. However, on the third day of exposure both types of cells had internalized significant numbers of micron-sized fluorescent polymer particles as shown in Figure 3(C,F). In order to confirm that the particles were inside the cells and they were not associated with the cell surface, the cells were visualized using confocal/deconvolution microscopy. After 24 h incubation with the particles the dural fibroblasts had taken up fluorescent micron-sized particles (Supporting Information, Movie 1) whereas the dural epithelial cells did not contain particles (Supporting information, Movie 2).

**Figure 4:**
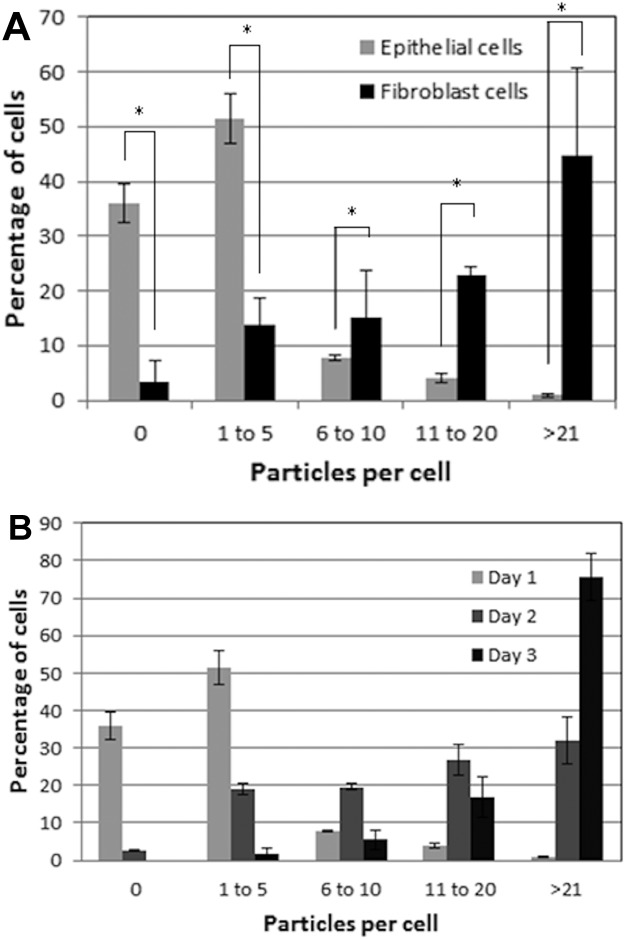
Cellular uptake of fluorescent microspheres (1 µm in diameter). (A) Percentage of dural fibroblasts (gray) and dural epithelial (black) cells that had phagocytosed a given number of particles after 24 h exposure. The results were analyzed by one-way ANOVA test and the MSD calculated using the T-method. A statistical difference (*p* < 0.05) in particle uptake between the two group of cells, is indicated by an asterisk (*) (B) Percentage of dural epithelial cells that had phagocytosed a given number of particles after 24 h (white), 2-day (gray), and 3-day (black) exposure. Data are expressed as the mean (*n* = 3) ± 95% confidence limits. [Color figure can be viewed in the online issue, which is available at http://wileyonlinelibrary.com.]

To further assess differences between epithelial and fibroblast cell uptake of micron sized particles, the number of particles per cell was quantified (Figure 4). There was a significant difference (*p* < 0.05) in the particle uptake between the two types of cells after 24 h of exposure. The majority of the dural epithelial cells contained no particles (∼35% of the cells) or 1 to 5 particles (∼50% of the cells). However, the dural fibroblasts had engulfed 11 to 20 particles (∼23 % of the cells) or more than 21 particles (∼45% of the cells). After 48 h of epithelial cell particle exposure, there was a gradual shift from 0 or 1–5 particles per cell to 6 to 10, 11 to 20, and more than 21 particles per cell. After 3 days when the majority of the cells had internalized more than 21 particles. Some images showed nanoparticles outside the cells but these were not count in the results.

**Figure 5:**
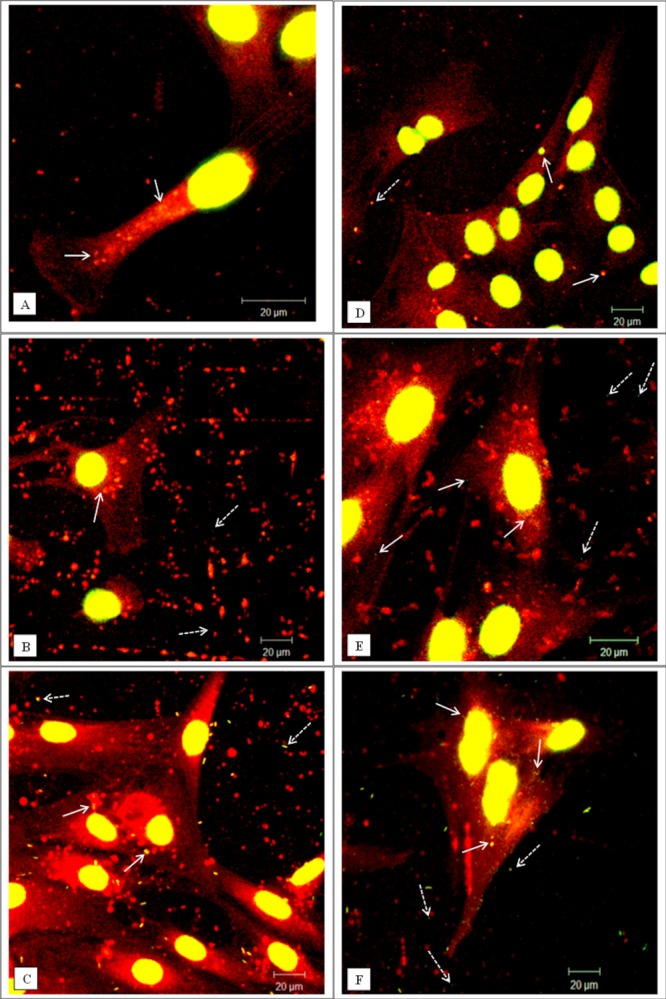
Representative confocal microscopy images of cells exposed to green fluorescent nano-particles (40 nm in diameter). (A–C) Dural epithelial cells. (D–F) Dural fibroblasts. (A, D) Cells exposed for 1 day. (B, E) Cells exposed for 2 days. (C, F) Cells exposed for 3 days. White arrows indicate particles internalised whereas white dashed arrows indicate particles outside from cells. After day 2 there is evidence of particle agglomeration. The actin filaments were stained with rhodamine phalloidin and the cell nuclei with sytox green. All the images were taken at 630× magnification. In total 300 cells were imaged (three replicates × 100) at each time point. [Color figure can be viewed in the online issue, which is available at wileyonlinelibrary.com.]

This pattern of nanoparticle uptake was different. Both epithelial and fibroblast cells contained high numbers of 40 nm sized particles after 24 h exposure (Figure 5). It was not possible determine the specific number of nanoparticles in each cell type since the confocal images were limited by resolution. Images taken at day 2 and 3 showed that both epithelial and fibroblast cells internalized high numbers of nanoparticles. Also, these particles after the second day had become agglomerates of variable size and number. This fact made it impossible to quantify the uptake of the nanoparticles from the two types of cells.

## DISCUSSION

The biological effects of wear particles generated in TDR in the dura mater pose a potential clinical problem. There is a need to understand particle interaction with dural cells. A porcine model was used for the isolation and characterization of dural cells for studies of the effects of model particles *in vitro*. Dural cells were used since this tissue acts as a protective membrane for the spinal cord, preventing infiltration of foreign bodies to the neural tissue.^23^

The porcine dura mater comprised a thin membranous structure containing fibroblastic cells with a layer of epithelial cells apically. The epithelial layer expressed VWF and fibronectin. VWF is a multimeric glycoprotein constitutively expressed by endothelial cells and stored in cytoplasmic granules called Weibel-Palade bodies 24. The role of VWF is related to the repair of damaged endothelia.^24^ The dural extracellular matrix was comprised of collagen I, II, and III. The porcine dural matrix was similar to the human dura which is primarily composed of collagen fibers interspersed with elastin fibers.^25^ The epithelial cells expressed E-cadherin, which is indicative of adherens junctions and desmoplakin, a marker for desmosomes. E-cadherins play a crucial role in physically connecting neighbouring epithelial cells, linking adherens junctions to the actin-myosin network, control vesicle transport and modulate cell polarity machinery. Also, they can play a role in maintaining more stringent barriers such as the blood brain barrier.^26^,^27^ This is the first report of the presence of E-cadherin in dural epithelial cells.

Immunohistochemical staining of the two cell types isolated from the dura mater revealed that the dural epithelial cells expressed VWF and CD31 exhibiting the same characteristics as typical endothelial cells.^28^,^29^ An important property of epithelial cells is their assembly into a physical and ion- and size-selective barrier separating tissues.^30^ In order to identify any potential barrier function of the cells they were analyzed for desmoplakin I&II and Glut1 expression. Desmoplakin is a component of desmosomes that attaches intermediate filaments on desmosomal plaques that are present in blood brain barrier.^31^ Glut1 has been shown to be selectively expressed in the apical and basolateral membrane of blood brain barrier.^32^ Dural epithelial cells expressed desmoplakin I&II, especially in regions of cell confluency, enforcing their role barrier function and protection of neural tissue. Dural fibroblasts did not express desmoplakin I&II. However, both cell types expressed Glut1 suggesting that both cell types utilized this glycoprotein to transport glucose into the cell. Amplified Glut1 expression may, however, have been due to exposure of the cells to serum growth factors in culture.^33^,^34^

Commercial polystyrene spheres were used to assess particle uptake by the cells since this enabled studies of homogenous particles of known size and number. These particles have been used to study particle translocation across a barrier^35^ and to determine the phagocytic ability of numerous cell types.^36^ In the case of micron-sized (1 µm) particles, the internalization was different between the two types of cells. The dural epithelial cells exposed to micron sized particles for 24 h, internalized either no particles or a very small number (<5 particles per cell). Dural fibroblasts internalized 11 to 20 micron particles or more than 20 particles in 24 h. There were some nanoparticles outside the cells but they were not count in the results. Differences between the capacity of different cell lines to internalise polystyrene microspheres and micron-size silica particles have been widely documented.^37^,^38^ Confocal and deconvolution microscopy, an established method for detection of particle internalization,^39^ confirmed the difference between dural fibroblasts and epithelial cell capacity to take up micron sized particles.

The internalization of nanometer sized particles (40 nm) was evident in both dural epithelial cells and fibroblasts but it was difficult to quantify the particle uptake due to the limited resolution of confocal microscopy as well as the fact that nanoparticle agglomeration occurred after the first 24 h. It is well known that nanoparticles tend to cluster, forming an agglomerated state and they may behave as larger particles depending on the size of the agglomerate.^47^ The ability to detect nanoparticle uptake by cells using confocal microscopy is well established for different materials.^40^–^42^ However, conventional confocal microscopy has many limitations for the quantification of nanoparticle uptake by cells and therefore modifications such as hyper-spectral confocal imaging and Raman confocal microscopy as well as flow cytometry have been developed.^43^–^45^ In addition, Raman spectroscopy methods can be used to detect metal nanoparticle signatures in living cells and can be used to follow the pattern of uptake without the need for fixation or staining of cells.^46^ This technique will be useful in future studies of the biological effects of clinically-relevant prosthetic CoCr nanoparticles on cells of the dura mater.

## CONCLUSION

This study is the first to analyze the components of the dural membrane in depth and enforces the hypothesis that the dura mater acts in defence against foreign body infiltration. Differences in the uptake of model particles between the different types of dural cells were demonstrated. Nanometer sized particles rapidly penetrated dural epithelial and fibroblast cells, whereas dural fibroblasts engulfed micron sized particles at a much higher rate than the dural epithelial cells. These findings give an initial insight into how the size of particles may determine their interaction with the cells of the dura mater. The dural epithelial cells may provide a transient barrier to micron-sized particles but not nanoparticles. It will be important to determine the biological consequences of exposure of the cells to clinically relevant particles generated by metal-on-metal and metal-on-polyethylene TDR.
